# Daytime-restricted feeding induces lean MAFLD in high-fat diet-fed mice by upregulating CD36-mediated lipid accumulation

**DOI:** 10.1016/j.jlr.2025.100853

**Published:** 2025-06-23

**Authors:** Zhenyu Wang, Mingyang Zhang, Miao Chen, Shuning Fu, Yang Zhang, Mengyue Chen, Xiong Z. Ruan, Yaxi Chen

**Affiliations:** 1 Centre for Lipid Research & Chongqing Key Laboratory of Metabolism on Lipid and Glucose, Key Laboratory of Molecular Biology for Infectious Diseases (Ministry of Education), Institute for Viral Hepatitis, Department of Infectious Diseases, The Second Affiliated Hospital, Chongqing Medical University, Chongqing, China; 2 John Moorhead Research Laboratory, Centre for Nephrology, University College London Medical School, Royal Free Campus, University College London, London, United Kingdom

**Keywords:** time-restricted feeding, dyslipidemia, liposomal nanoparticles, hepatology, lipogenesis

## Abstract

Time-restricted feeding (TRF) may aid in weight loss and improve metabolic health; however, its long-term effects and applicability to all individuals remain unclear. This study investigated the impact of different dietary patterns on hepatic metabolism by subjecting mice to either a normal chow diet or a high-fat diet, allowing for ad libitum feeding, daytime restrictive feeding (DRF), or nighttime restrictive feeding (NRF). Using metabolic cages to assess energy intake, we found that the fuel utilization rhythms of DRF mice were disrupted compared to ad libitum-fed mice. Mice on normal chow DRF exhibited only dyslipidemia, while those on high-fat DRF developed lean metabolic dysfunction-associated fatty liver disease (MAFLD), characterized by more pronounced dyslipidemia, weight loss, and hepatic lipid accumulation. RNA seq revealed that CD36 plays a crucial role in the development of lean MAFLD induced by high-fat DRF by inhibiting AMPK phosphorylation, disrupting the balance between lipogenesis and oxidation. Mechanistic validation was performed in CD36 liver-specific knockout mice and Liposomal nanoparticle injection models. These findings provide new insights into the potential mechanisms linking feeding patterns to lean MAFLD. Additionally, CD36 emerges as a potential therapeutic target for high-fat-induced lean MAFLD. Clarifying the relationship between DRF and lean MAFLD may inform guidelines for specific populations, such as individuals practicing intermittent fasting or those working night shifts, while also suggesting potential therapeutic strategies for clinical management.

MAFLD, which may progress to Metabolic Dysfunction-Associated Steatohepatitis (MASH) and cirrhosis (1, 2, 3), has emerged as a major public health concern globally, with a prevalence of approximately 25% worldwide (4, 5, 6). Although obesity plays a pivotal role in MAFLD (7), approximately 10%–20% (8) of individuals with MAFLD who are not overweight or obese (BMI < 25 kg/m2, or BMI < 23 kg/m2 in Asians) have “Lean MAFLD”. But clearly, lean MAFLD is a neglected and underappreciated subtype. Lean MAFLD is defined as a “metabolically unhealthy normal weight” MAFLD, which is characterized by a decrease in subcutaneous fat and ectopic fat deposition in the liver (9). It’s the major cause of cryptogenic liver disease (10), and lean individuals with MAFLD have a higher risk of impaired glucose tolerance, hypertension, metabolic syndrome, and cardiovascular morbidity (11). Studies highlight that lean MAFLD is a complicated phenotype of MAFLD (12); moreover, the stealthiness of this disease makes it much difficult to improve clinical outcomes, thus, much more research is needed to figure out the pathogenesis.

The swift rise in the prevalence of MAFLD is occurring rapidly worldwide, which can be partly attributed to a change in lifestyle, such as different dietary composition or irregular meal patterns (6). As yet, most drugs in clinical practice lack effectiveness for MAFLD. In this light, time-restricted feeding (13) or other forms of dietary interventions, such as intermittent fasting (IF) (14) or fasting-mimicking diet (15), have gained increased significance as viable interventions against obesity and metabolic diseases. Nighttime-restricted feeding (16) has been demonstrated to ameliorate metabolic disorders in rodent models. However, emerging evidence from studies involving night shift workers (17) and individuals observing Ramadan (18) suggests that daytime-restricted feeding in nocturnal mice (which corresponds to daytime fasting and nighttime eating in diurnal humans) may be linked to an elevated risk of metabolic diseases. The connection between eating patterns and lean MAFLD remains ambiguous, and to date, there have been no reported studies on the relationship between TRF and lean MAFLD. Therefore, eating patterns may be a new insight to explore the mechanism of lean MAFLD.

CD36, a transmembrane glycoprotein involved in fatty acid uptake and storage (19), plays a significant role in maintaining the lipid metabolic balance within cells. Hereditary CD36 deficiency is common in Asian and African countries, accounting for about 3%–10% of the population (20, 21). These people are more likely to exhibit metabolic dysfunction and even metabolic syndrome, which can lead to a series of metabolic-related diseases. Studies have shown that CD36 expression is upregulated in the livers of patients with MAFLD (22, 23). Our previous study found that increased CD36 expression has been linked to exacerbated steatosis by promoting hepatic fatty acid uptake and triglyceride storage (24). On the one hand, CD36 is coupled to Insig2 to eliminate the interaction between Insig2 and the SCAP-SREBP complex, causing an increase in lipid synthesis in the liver (22). On the other hand, CD36 plays a negative role in regulating autophagic degradation of lipid droplets via AMP-activated protein kinase (AMPK) dependent pathway (23, 25), and once AMPK is activated by phosphorylation, it initiates a range of metabolic pathways, including promoting fatty acid oxidation and inhibiting fatty acid synthesis. The role of CD36 in obesity-related MAFLD has been extensively studied, and it can even be a therapeutic target. This raises the possibility that CD36 may also make sense in improving lean MAFLD.

Here, we reported that the DRF regimen may increase the risk of lipid metabolism disorders. furthermore, high-fat DRF upregulated CD36 expression, influenced AMPK-related lipid synthesis and oxidation, thus significantly enhanced disease susceptibility and led to lean MAFLD. We identified that the inhibition of CD36 expression in hepatocytes may serve as a potential therapeutic target for dietary factor-induced lean MAFLD. By elucidating these underlying mechanisms, we can implement targeted interventions aimed at modulating CD36 expression, ultimately preventing and managing lean MAFLD associated with dietary factors. Furthermore, this strategy may also be beneficial in addressing metabolic disorders caused by dietary factors in populations such as night shift workers and those practicing intermittent fasting, thereby proposing a potential treatment approach for their metabolic diseases.

## Materials and Methods

### Animal care and strains

CD36 knockout (*Cd36*^−/−^) mice created on a C57BL/6J background were kindly provided by Dr Maria Febbraio (Lerner Research Institute). Alb-cre^+/−^ mice were obtained from Shanghai Research Center for Model Organisms (China). Cd36^f/f^ mice, in which the exon 5 of the CD36 allele was flanked with loxP recombination sites, were generated and crossed with either Alb-cre^+/−^ mice to generate hepatocyte-specific CD36 knockout mice. Cd36^f/f^ mice were identified by tail genomic DNA analysis with primer F specific to the upstream LoxP locus (5′-TCCCTTGAATTGGCCAACTTTG-3′) and primer R (5′-ACTGCCTGTGAGAACTTCTCAA−3′), an antisense specific to the downstream LoxP locus (22, 26). CD36 deletion was verified by genotyping of tail DNA (PCR), along with mRNA (RT-qPCR) and protein (Western blot) analysis in liver tissues. Control animals were Cre-negative, floxed Cd36 littermates to ensure comparability. All comparisons between Cd36 LKO and control mice were performed using age- and sex-matched animals from the same breeding cohort. Male, 8-week-old mice as described above were used for this study. Animal care and experimental procedures were performed with approval from the Ethics Committee of the Second Affiliated Hospital of Chongqing Medical University. And the investigation conforms with the Guidelines for the Care and Use of Laboratory Animals published by the US National Institutes of Health (NIH Publication No. 85-23, revised 1996).

### Metabolic profiling studies

Experimental mice were housed for 4–5 days in TSE PhenoMaster metabolic cages (TSE Systems) to allow acclimation to the new caging environment. Food and water intake, movement distance, physical activity, oxygen consumption (VO_2_), carbon dioxide production (VCO_2_), respiratory exchange ratio (RER), and heat production were then simultaneously measured during 48-h metabolic profiling studies. Food and water were provided ad libitum via appropriate devices, and all mice were maintained under a 12-h light/dark cycle.

### Time-restricted feeding and sample collection

Eight-week-old C57BL/6J male mice were fed normal chow diet (NCD, D12450B, Research Diets) containing 10 kcal% fat or an HFD (D12492, Research Diets) containing 60 kcal% fat for 16 weeks under AL feeding, daytime-restricted feeding or nighttime-restricted feeding. Zeitgeber time zero (ZT0) was referred to lights on at 6:00 AM. The DRF group had access to food for 12h from ZT1 to ZT13. The NRF group had access to food for 12h from ZT13 to ZT25 (ZT1). All mice were returned to ad libitum feeding two days prior to the end of the final fasting cycle and were subsequently euthanized at ZT8 (2:00 PM) in a fed state to standardize the sampling time point across groups.

### Glucose tolerance tests (GTTs) and insulin tolerance tests (ITTs)

GTTs were performed in 12-h-fasted mice following an intraperitoneal injection of glucose (1 g/kg body weight), and ITTs were performed in 4-h-fasted mice following an intraperitoneal injection of insulin (0.7 units/kg body weight). Blood glucose levels from tail vein blood were measured at 0, 15, 30, 60, 90, and 120 min with an ACCU-CHEK Advantage glucometer (Roche Diagnostics).

### Serum biochemistry analysis

The levels of non-esterified free fatty acid (NEFA), triglycerides (TG), total cholesterol (TC), high density lipoprotein (HDL), low density lipoprotein (LDL), aspartate aminotransferase (AST), and alanine aminotransferase (ALT) in the serum samples were analyzed by an automatic biochemistry analyzer. Serum insulin levels were analyzed separately using an insulin kit (CK-20353m, Ding Guo), according to the manufacturer’s protocols.

### Histological analysis

The liver was fixed in 4% paraformaldehyde in PBS. Histological changes were examined by hematoxylin & eosin (H&E) stain. Lipid accumulation in the liver was analyzed by Oil Red O staining on the frozen liver tissues. Micrographs were captured using an automated whole-slide scanning device (3DHISTECH).

### Oil-Red-O staining

Liver specimens were snap-frozen in optimal cutting temperature (OCT) compound and stored at −80°C until sectioning. Consecutive 8–10 μm cryosections were mounted on pre-cleaned slides, fixed in 10% neutral buffered formalin (10 min, RT), and rinsed with 60% isopropanol. Tissue sections were stained with freshly prepared 0.5% Oil Red O (in 60% isopropanol, 15 min, RT), washed, and counterstained with hematoxylin before aqueous mounting. High-resolution images were acquired using a Zeiss Axio Imager microscope under standardized illumination.

### Immunohistochemistry

Fixed and paraffin-embedded liver sections were deparaffinized and incubated in Target Retrieval Solution (Dako) buffer at 95°C for 35 min for antigen retrieval and then incubated overnight at 4°C with the primary antibodies. Biotinylated secondary antibodies (Pharmin gen) were added and incubated for 20 min at room temperature. Streptavidin-horseradish peroxidase (Pharmingen) was added, and after 30 min, the sections were developed with 3,3′-diaminobenzidine (DAB) substrate (Vector Laboratories) and counterstained with hematoxylin.

### RNA isolation, real-time quantitative PCR (Q-PCR) analysis, and RNA sequence

Liver tissues or cells were lysed in RNAiso Plus (9108, Takara, Japan) to extract total RNA according to the manufacturer’s protocols. cDNA was synthesized by a Prime Script RT reagent kit (DRR037A). Q-PCR was performed using the SYBR Green PCR Mix kit (Takara, Japan) and the CFX connect real-time system (Bio-Rad). Gene expression levels were normalized to β-actin, and relative levels were compared to control samples using the 2-DDCt method. The specific primer sequences used for real-time PCR are listed in supplemental Tables S1 and S2.

For transcriptome profiling, ALF, DRF, and NRF mice were fed either HFD or NCD for 16 weeks, after which liver mRNA sequencing was performed using the Illumina HiSeq 2000 platform (Majorbio Biotech). Raw sequencing data were processed and analyzed on the Majorbio I-Sanger Cloud Platform (www.i-sanger.com). The raw RNA-seq data have been deposited in the NCBI Sequence Read Archive (SRA) under accession number PRJNA1234793.

### Western blot analysis

Proteins from tissue and cell lysates were extracted using RIPA buffer. A total of 20–50 μg of protein was used for Western blot. Antibodies were pre-validated by molecular mass using positive control samples. The antibody used in this experiment, and the dilution concentration, is listed in supplemental Tables S1.

### Preparation and characterization of liposomal nanoparticles

The solvent in the phospholipid solution was evaporated in a current of nitrogen to obtain a thin layer of phospholipids. An aqueous dispersion of the Si-RNA complex in phosphate-buffered saline (PBS) of pH 7.4 was added to the thin film and stirred constantly for 30 min at 60°C. The solution was extruded through polycarbonate membranes with a pore diameter of 200 nm for 10 cycles to obtain uniformly sized liposomes and was freeze–dried. Mice were first fed HFD-DRF for 12 weeks to establish lean MAFLD. Following this induction phase, the animals received a single tail vein injection of siRNA at a dose of 0.4 μg/g body weight (delivered in 5 μl/g volume). Immediately after siRNA administration, all mice were switched to NCD ad libitum for 10 days before euthanasia.

### Statistical analysis

The difference between two groups was statistically analyzed by Student’s *t* test. The differences among three or more groups were analyzed by one-way analysis of variance (ANOVA) followed by Tukey’s multiple comparisons test. Statistical significance was defined as ∗*P* < 0.05, ∗∗*P* < 0.01, and ∗∗∗*P* < 0.001.

## Results

### DRF causes an increased susceptibility to lipid metabolism disorders in mice

**Fig. 1:**
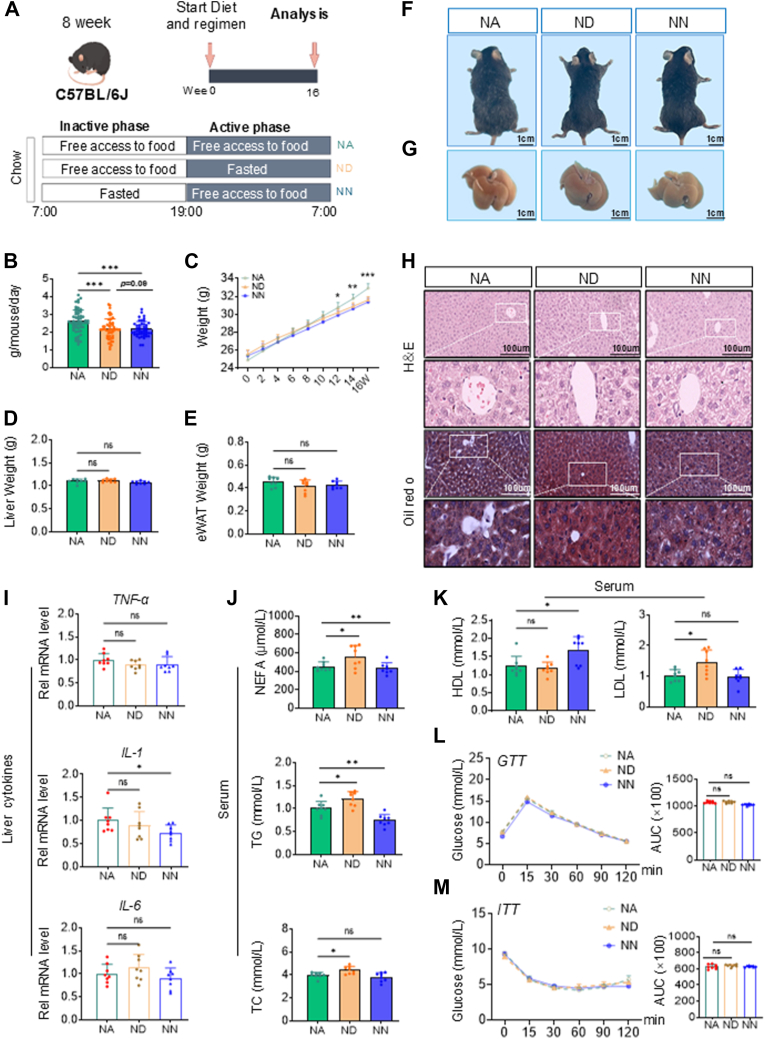
A: Schematic outline of six feeding regimens used in this study: NCD-ad libitum (NA), NCD-DRF (ND), NCD-NRF (NN). B: Daily intake of ad libitum diet and TRF (DRF and NRF) mice in NCD background (n = 8). C: Body weight of ad libitum diet and TRF (DRF and NRF) mice in NCD background (n = 8). D: Liver weight of mice in the NCD background. E: e-WAT weight of mice in NCD background. F, G: Representative pictures of mice and liver sections were shown. H: Representative pictures of HE-staining and ORO-staining in mice liver sections were shown. I: Relative mRNA levels of IL-1β, IL-6, and TNF-α in mice livers (n = 8). J, K: Serum levels of non-esterified fatty acids (NEFA), triglyceride (TG), total cholesterol (TC), high-density lipoprotein (HDL), and *low*-density lipoprotein (LDL) in mice (n = 8). L, M: Glucose tolerance tests (GTTs) and insulin tolerance tests (ITTs) in mice. Quantification of the area under the curve (AUC) was shown on the right (n = 8). Statistical data were assessed using 1-way ANOVA with Tukey's multiple comparisons test. Data are presented as mean ± SEM. ∗*P* < 0.05; ∗∗*P* < 0.01; ∗∗∗*P* < 0.001.

To investigate the effects of different dietary times on metabolism, we used three different dietary models: (1) Normal chow diet -Ad libitum feeding (NA), (2) Normal chow diet -DRF (ND), (3) Normal chow diet -NRF (NN). 8-week-old male C57BL/6J mice were fed a chow diet (NCD) with time-restricted (DRF and NRF) access to food (12h free access to food daily) for 16 weeks. Because mice are primarily nocturnal, DRF mice had free access to food and water between 7 am and 7 pm, and NRF mice had ad libitum access to food and water from 7 pm to 7 am. (Fig. 1A). The control groups were given ad libitum access to food and water.

**Figure S1:**
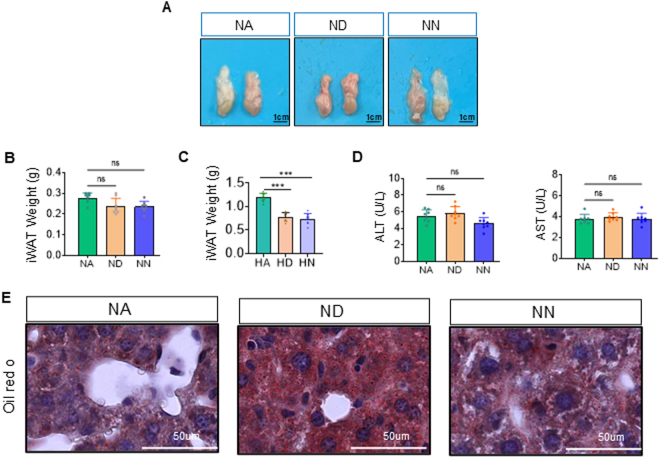
(A) Representative pictures of e-WAT are shown. (B) i-WAT weight of mice in NCD background. (C) i-WAT weight of mice in HFD background. (D) The serum levels of ALT and AST in the mice (n=8). Statistical data were assessed using 1-way ANOVA with Tukey's multiple comparisons test. Data are presented as mean ± SEM. ∗P < 0.05; ∗∗P < 0.01; ∗∗∗P < 0.001.

Because of the reduction in feeding time, the mice consumed a smaller amount of food than their ad libitum cohorts (Fig. 1B). From 12 weeks onward, TRF (DRF and NRF) significantly prevented body weight gain in mice (Fig. 1C). After the 16-week feeding trial, we evaluated the weights of the liver and selected white adipose tissues in mice from all experimental groups (Fig. 1D, E and supplemental Fig. S1B) and documented the findings with photographic records (Fig. 1F, G and supplemental Fig. S1A). All mice on TRF weighed less than their respective littermate controls. Notably, although chow-fed TRF mice showed an approximate 2 g body weight difference compared to controls, no significant differences were observed in eWAT and iWAT weights between these groups. However, we observed a trend toward higher iWAT weight in control mice (approximately 0.1 g greater than TRF mice, *P* = 0.07). This subtle difference suggests that the observed body weight variation may primarily stem from differences in other organs or lean mass. Histological analysis of liver sections showed that 16 consecutive weeks of ND did not cause liver injury and lipid accumulation in mice (Fig. 1H, I and supplemental Fig. S1D). Interestingly, we found that the ND mice had increased serum low-density lipoprotein (LDL), triglycerides (TG), and total cholesterol (TC) compared to the NA group, while the NN mice (27, 28) did not (Fig. 1J, K). To the opposite, there were no significant changes in glucose tolerance and insulin sensitivity in ND mice (Fig. 1L, M). The above results suggested that DRF leads to increased susceptibility to lipid metabolism disorders in mice compared to ad libitum diet and NRF, which is due to altered eating timing.

### Normal chow DRF disrupts diurnal rhythms in fuel utilization

**Fig. 2:**
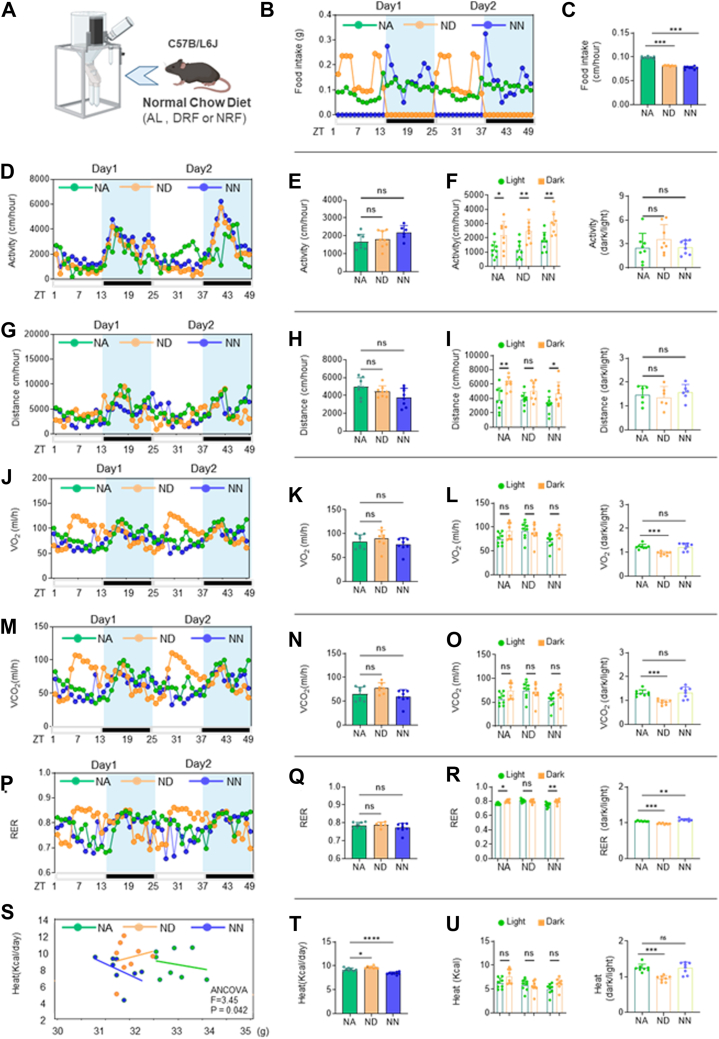
A: Schematic diagram of a mouse in a metabolic cage (TSE system). B, C: Hourly food intakes in the light/dark cycle and average hourly activity of ad libitum and TRF (DRF and NRF) mice on NCD (n = 6–8/group, recorded for 2 days). D–F: Hourly locomotor activity in the light/dark cycle, average hourly activity, and dark/light activity ratio of ad libitum and TRF (DRF and NRF) mice on NCD (n = 6–8/group, recorded for 2 days). G–I: Hourly movement distance in the light/dark cycle, average hourly movement distance, and dark/light movement distance ratio of ad libitum and TRF (DRF and NRF) mice on NCD (n = 6–8/group, recorded for 2 days). J–L: Hourly oxygen (O_2_) consumption in the light/dark cycle, average hourly O_2_ consumption, and dark/light O_2_ consumption ratio in ad libitum and TRF mice (n = 6–8/group, recorded for 2 days). M–O: Hourly carbon dioxide (CO_2_) production in the light/dark cycle, average hourly CO_2_ production, and dark/light CO_2_ production ratio in ad libitum and TRF mice (n = 6–8/group, recorded for 2 days). P–R: Hourly respiratory exchange ratio (RER) in the light/dark cycle, average hourly RER, and dark/light RER ratio in ad libitum and TRF mice (n = 6–8/group, recorded for 2 days). S–U: Covariance Analysis of Body Weight and Heat Production in Ad Libitum versus TRF Mice: Weight-Adjusted Heat Production, Hourly Heat Production in Light/Dark Cycles, and Dark/Light Heat Production Ratio (n = 6–8/group, recorded for 2 days). Statistical data were analyzed using 1-way ANOVA with Tukey's multiple comparisons test, or analysis of covariance (ANCOVA) with body weight as the covariate when appropriate. Data are presented as mean ± SEM. ∗*P* < 0.05; ∗∗*P* < 0.01; ∗∗∗*P* < 0.001.

The role of DRF in reducing weight gain but enhancing dyslipidemia prompted us to test whether DRF affected daily patterns of whole-body fuel utilization. NA, ND, and NN mice were analyzed in metabolic cages (TSE Systems) for 4–5 days (after 48 h acclimation) after undergoing 14–15 weeks of the indicated feeding regimens (Fig. 2A). Intriguingly, time feeding restriction (Fig. 2B, C) induced significant circadian metabolic alterations independent of locomotor activity (Fig. 2D–I). Specifically, we observed that ND mice exhibited marked circadian rhythm changes in oxygen consumption (VO_2_) (Fig. 2J–L) and carbon dioxide elimination (VCO_2_) (Fig. 2M–O) without any alterations in locomotor activity or movement distance. These changes were particularly manifested as increased daytime oxygen consumption (Fig. 2L) and CO_2_ production (Fig. 2O). Furthermore, while both NA and NN mice maintained a relatively stable respiratory exchange ratio (RER) throughout the day-night cycle (Fig. 2P–R), indicating consistent metabolic substrate utilization, ND mice displayed pronounced diurnal RER rhythms. This suggests frequent switching between fuel sources across day-night cycles. The RER (16) in ND mice increased during daytime, reflecting feeding behavior and subsequent carbohydrate utilization, while decreasing at night, consistent with lipid oxidation during fasting periods. To investigate the differences in heat production induced by different feeding times and control for the effect of body weight, a covariance analysis (ANCOVA) was used, with body weight as the covariate and heat production as the dependent variable. The ANCOVA results showed that the covariate body weight had a significant effect on heat production (Fig. 2S), indicating that the variation caused by body weight needs to be controlled during intergroup comparisons. The adjusted mean heat production of the DRF group was significantly higher than that of other groups (Fig. 2T). Feeding time intervention significantly altered the thermogenic rhythm in mice, especially manifested as enhanced daytime thermogenesis (Fig. 2U).

These findings collectively suggest that daytime restricted feeding disrupts the normal fuel utilization rhythm in mice fed a standard diet, highlighting the critical impact of meal timing on metabolic regulation.

### Nonobese metabolic syndrome induced by high-fat DRF features lean MAFLD

In previous results, we found that Normal chow DRF led to an increased risk of lipid metabolic diseases in mice, despite the absence of significant pathological damage. However, some studies have found that people with circadian rhythm disorders are more likely to choose unhealthy foods than those who get enough sleep, such as high-fat meals (29, 30, 31). To evaluate the effect of the DRF on metabolism in a high-nutrient state, we fed mice with a high-fat diet (60% energy from fat, HFD) at the same time as time-restricted feeding (32). We used three other different dietary models: (1) High-fat diet -Ad libitum feeding (HA), (2) High-fat diet -DRF (HD), (3) High-fat diet -NRF (HN). The feeding time for each group was the same as in the NCD background.

**Fig. 3:**
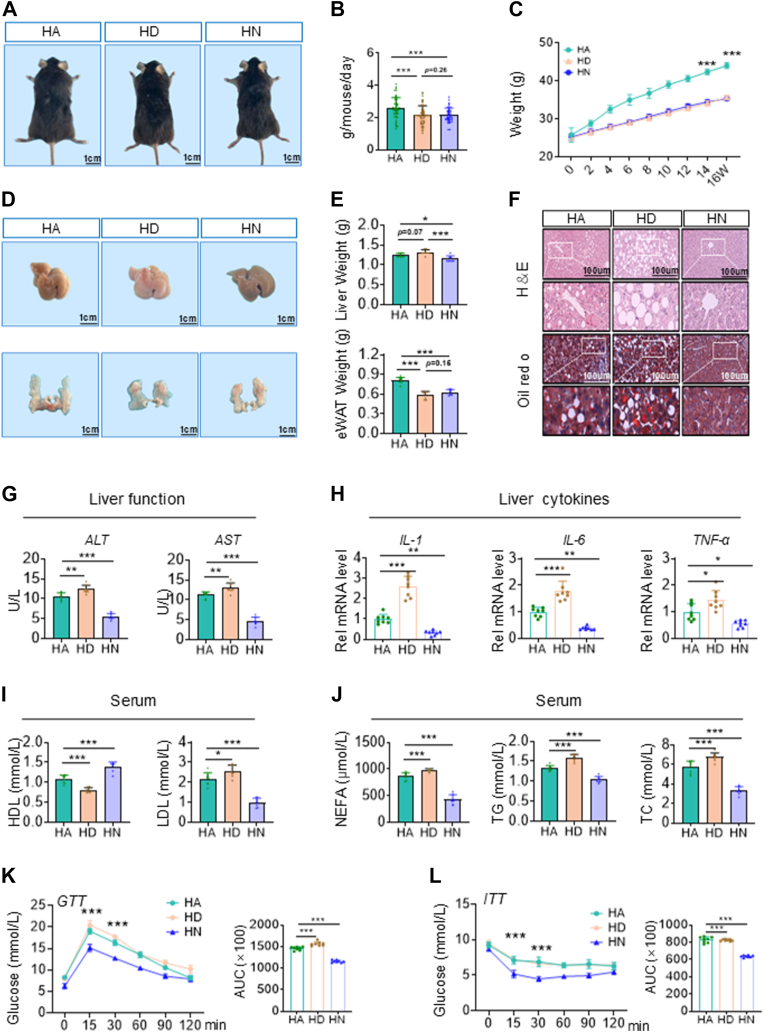
A: Representative images of mice. B: Daily intake of ad libitum diet and TRF (DRF and NRF) mice in HFD background (n = 8). C: Body weight of mice. D: Representative pictures of mice and liver sections were shown. E: Weight of mice liver and e-WAT (n = 8). F: Representative pictures of HE-staining and ORO-staining in mice liver sections were shown. G: The serum levels of ALT and AST in the mice (n = 8). (H) Relative mRNA levels of IL-1β, IL-6 and TNF-α in mice livers (n = 8). I, J: Serum levels of NEFA, TG, TC, HDL and LDL in mice (n = 8). K, L: GTTs and ITTs in mice. Quantification of the area under the curve (AUC) was shown on the *right* (n = 8). Statistical data were assessed using 1-way ANOVA with Tukey's multiple comparisons test. Data are presented as mean ± SEM. ∗*P* < 0.05; ∗∗*P* < 0.01; ∗∗∗*P* < 0.001.

In HFD background, our results found that mice ate less and lost weight under the DRF and NRF compared with an ad libitum diet (Fig. 3A, B). From 4 weeks onwards, TRF significantly prevented the weight gain of mice (Fig. 3C). This is earlier than in the NCD context. Most studies argued that weight loss is always accompanied by a reduction in obesity (33, 34, 35). Interestingly, we found that HD mice had a decrease in subcutaneous fat but an increase in liver weight (Fig. 3D, E and supplemental Fig. S1C). Notably, these differences were more pronounced compared to those observed under NCD conditions. The histology analysis of liver sections showed an increased hepatic fat accumulation in HD, but not in HN (27, 28) mice. Oil red O staining of liver showed more lipid droplets in HD mice compared with HA mice (Fig. 3F). Liver steatosis was observed in HD mice, accompanied by severe metabolic disturbances. While serum levels of alanine aminotransferase (ALT) and aspartate aminotransferase (AST) were elevated in HD mice compared to controls (Fig. 3G), they remained within the normal range for healthy mice, suggesting early metabolic stress rather than severe hepatocellular injury. The progression to more severe liver damage may require a longer duration of exposure (36). And the expression of proinflammatory cytokines *Tnf-α, Il6, and Il1β* (37, 38)—known contributors to MASH progression—was modestly but significantly increased in HD mice (Fig. 3H). These findings indicate that high-fat DRF, while promoting weight loss, exacerbates hepatic lipid accumulation and may initiate early metabolic liver dysfunction. Serological analysis revealed pronounced dyslipidemia in the HD group, with significant increases in TC, TG, NEFA, and LDL, alongside reduced HDL levels compared to the HA group (Fig. 3I, J). Furthermore, glucose tolerance and insulin sensitivity were impaired in HD mice, whereas the HN group exhibited protective effects against HFD-induced metabolic disruption (Fig. 3K, L). Importantly, since insulin sensitivity remained unchanged in NCD-fed DRF mice, we propose that dyslipidemia precedes insulin resistance in this model. Collectively, these data suggest that high-fat DRF induces a non-obese metabolic syndrome characterized by lean MAFLD, hyperlipidemia, and glucose intolerance.

### High-fat DRF also caused rhythmic changes in fuel utilization in mice

**Fig. 4:**
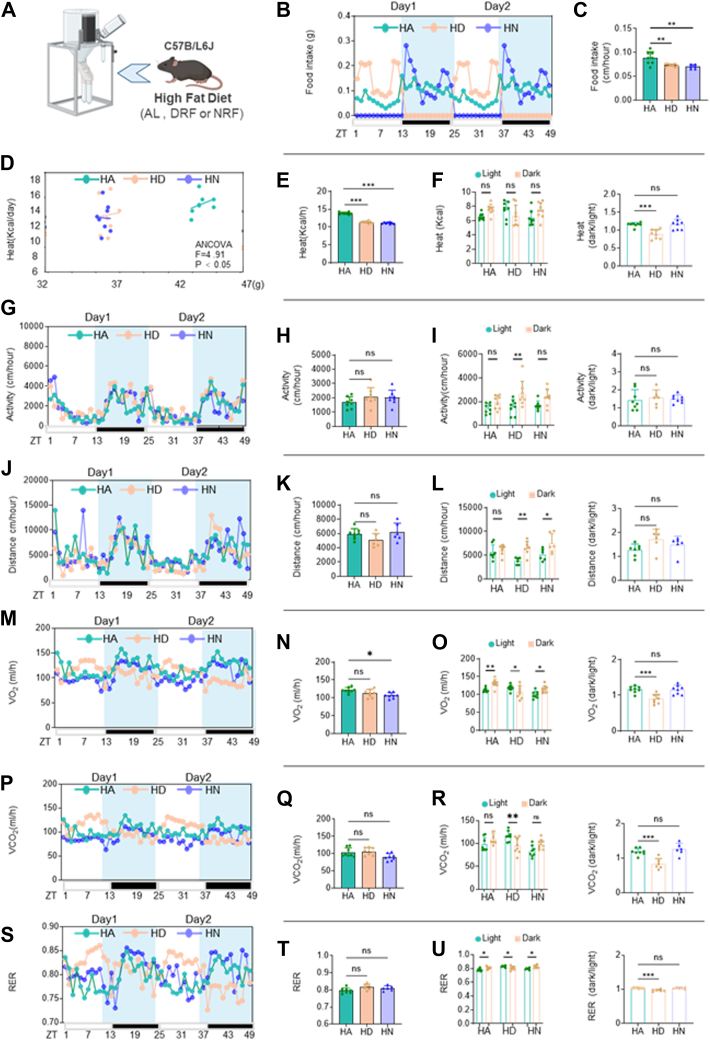
A: Schematic diagram of a mouse in a metabolic cage (TSE system). B, C: Food intakes per hour in the light/dark cycle, average activity per hour of ad libitum and TRF (DRF and NRF) mice in HFD (n = 6–8/group, recorded for 2 days). D–F: Covariance Analysis of Body Weight and Heat Production in Ad Libitum versus TRF Mice: Weight-Adjusted Heat Production, Hourly Heat Production in Light/Dark Cycles, and Dark/Light Heat Production Ratio (n = 6–8/group, recorded for 2 days). G–I: Locomotor activity per hour in the light/dark cycle, average activity per hour, and dark/light activity ratio of ad libitum and TRF (DRF and NRF) mice in HFD (n = 6–8/group, recorded for 2 days). J–L: Movement distance per hour in the light/dark cycle, average movement distance per hour, and dark/light movement distance ratio of ad libitum and TRF (DRF and NRF) mice in HFD (n = 6–8/group, recorded for 2 days). M–O: O_2_ consumption per hour in the light/dark cycle, average O_2_ consumption per hour, and dark/light O_2_ consumption ratio in ad libitum and TRF mice (n = 6–8/group, recorded for 2 days). P–R: CO_2_ production per hour in the light/dark cycle, average CO_2_ production per hour, and dark/light CO_2_ production ratio in ad libitum and TRF mice (n = 6–8/group, recorded for 2 days). S–U: RER per hour in the light/dark cycle, average RER per hour, and dark/light RER ratio in ad libitum and TRF mice (n = 6–8/group, recorded for 2 days). Statistical data were assessed using 1-way ANOVA with Tukey's multiple comparisons test, or ANCOVA with body weight as the covariate when appropriate. Data are presented as mean ± SEM. ∗*P* < 0.05; ∗∗*P* < 0.01; ∗∗∗*P* < 0.001.

To inquired about the effects of high-fat DRF on behavior and metabolism in mice. Same as the NCD pattern, HA, HD, and HN mice were analyzed in metabolic cages for 4–5 days after undergoing 14–15 weeks of the indicated feeding regimens (Fig. 4A). Notably, we observed feeding patterns strikingly similar to those in the NCD model: HD mice concentrated their caloric intake within three hours after feeding initiation and three hours before fasting onset, while HN mice maintained more evenly distributed food consumption throughout their 12-h feeding window. (Fig. 4B, C). This feeding pattern (potentially resembling binge-eating behavior) may contribute to exacerbated hepatic steatosis (39).

To explore the differences in energy expenditure induced by different feeding times under an HFD background and control for the effect of body weight, ANCOVA was used, with body weight as the covariate and energy expenditure as the dependent variable. The ANCOVA results showed that the covariate body weight had a significant effect on energy expenditure (Fig. 4D). Notably, the adjusted mean energy expenditure of the TRF group was significantly lower than that of the control group (Fig. 4E), which showed a distinct difference from the results under a NCD background (where the DRF group had higher energy expenditure than the control group). Interestingly, similar to the NCD paradigm, HFD mice again exhibited altered thermogenic rhythms characterized by increased daytime thermogenesis (Fig. 4F). Importantly, while locomotor activity (Fig. 4G–I) and movement distance (Fig. 4J–L) remained unchanged in HD mice, we detected significant circadian alterations in oxygen consumption (Fig. 4M, O) and carbon dioxide elimination (Fig. 4P–R) despite conserved total energy expenditure. These findings suggest that high-fat DRF disrupts the circadian regulation of energy metabolism, potentially underlying the development of lean MAFLD in this model. Further examination of whole-body fuel utilization patterns revealed circadian disturbances in substrate oxidation analogous to the NCD model (Fig. 4S–U). Collectively, these results demonstrate that across different dietary compositions, DRF induces intrinsic circadian reprogramming of systemic fuel metabolism independent of locomotor activity.

### High-fat DRF disrupted the balance of lipid synthesis and oxidation in the liver

From metabolic cage monitoring, we know that fuel utilization in DRF mice exhibits violent circadian rhythms. This may lead to a reduction in subcutaneous fat and ectopic fat deposits in the liver. To further explore the potential mechanisms for a decrease in subcutaneous fat and ectopic fat deposition in the liver in high-fat DRF mice, we performed RNA sequence analysis of the livers from TRF mice. For the NCD group, we first identified differentially expressed genes (DEGs) between DRF and ad libitum-fed mice as well as between NRF and ad libitum-fed mice. These DEGs were visualized using Venn diagrams to identify overlapping and unique gene sets.

**Fig. 5:**
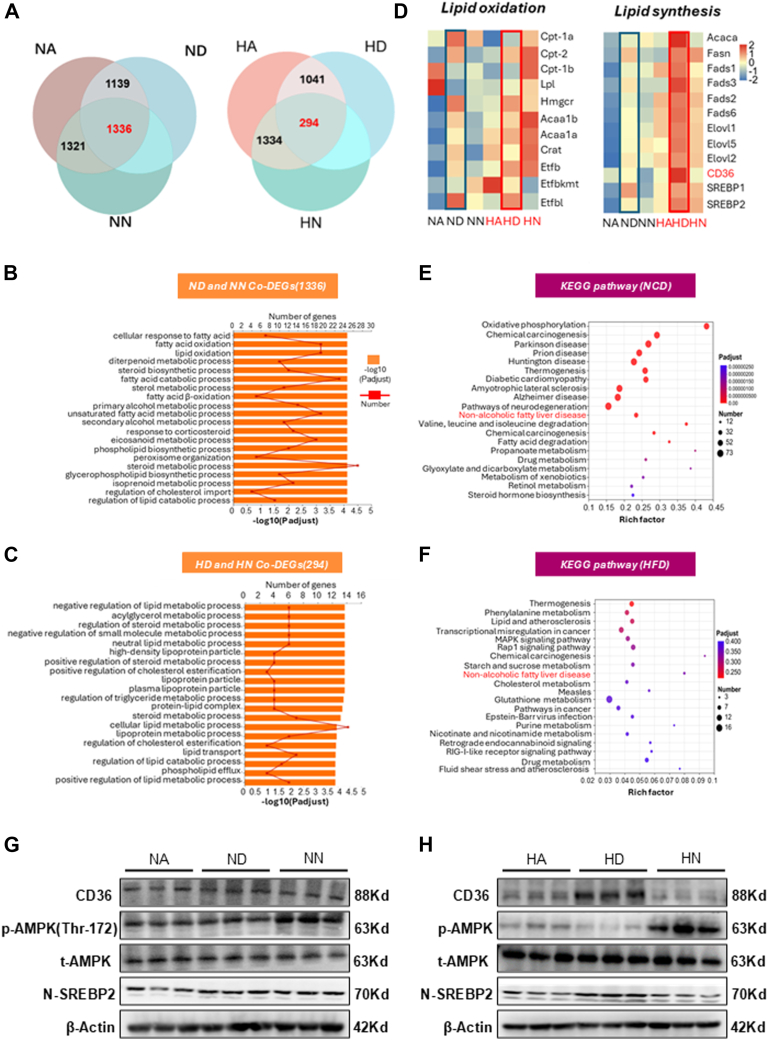
A: Transcripts of ad libitum and TRF (DRF and NRF) mice altered in the context of NCD and HFD (n = 4). B, C: *Top* 20 biological process gene ontology (GO) terms enriched by differentially expressed genes (DEGs) in ad libitum and TRF mice. D, E: Heatmap representation of genes involved in lipid oxidation and lipid synthesis. The gradual color ranged from *pink* to *blue* represented the mRNA expression changing from up-regulation to down-regulation. E, F: Top 20 KEGG pathways enriched by differentially expressed genes (DEGs) in ad libitum and TRF mice. G, H: The protein levels of CD36, p-AMPK, t-AMPK and Nuclear-SREBP2 (N-SREBP2) in livers from the ad libitum and TRF mice. Statistical data were assessed using 1-way ANOVA with Tukey's multiple comparisons test. Data are presented as mean ± SEM. ∗*P* < 0.05; ∗∗*P* < 0.01; ∗∗∗*P* < 0.001.

In the NCD group, we first identified differentially expressed genes (DEGs) among the ALF, DRF, and NRF groups to isolate genes specifically altered by TRF and exclude genes affected by non-TRF factors (Fig. 5A). The same analytical approach was applied to the HFD group to identify TRF-specific DEGs under high-fat diet conditions (Fig. 5A). A total of 1,336 DEGs were identified in the NCD group, and 294 DEGs were identified in the HFD group (Fig. 5A). We conducted GO analysis on these TRF-specifically altered genes.

**Figure S2:**
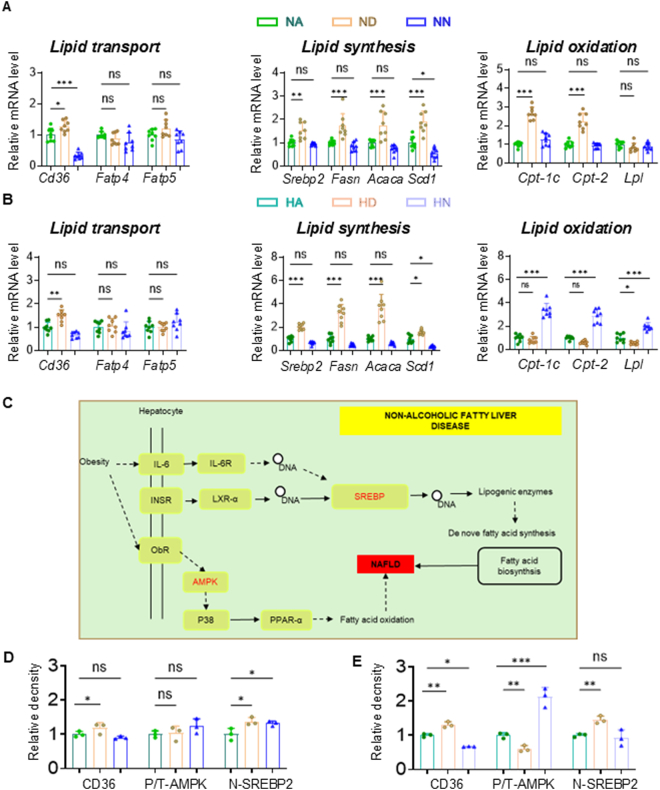
(A-B) Relative mRNA levels of lipid transport (Cd36, Fatp4, Fatp5), lipid synthesis (Srebp2, Fasn, Acaca, Scd1) and lipid oxidation (Cpt-1, Cpt2, Lpl) in mice liver (n=6-8). (C) Schematic diagram of key factors of the non-alcoholic fatty liver disease pathway (from the transcriptome database). (D-E) Relative densities of CD36, AMPK and SREBP2proteins in Figure 5E. Statistical data were assessed using 1-way ANOVA with Tukey’s multiple comparisons test. Data are presented as mean ± SEM. ∗P < 0.05; ∗∗P < 0.01; ∗∗∗P < 0.001.

Through GO analysis, we found these DEGs are enriched in lipid metabolic-related pathways (Fig. 5B, C). Of note, CD36 expression is significantly elevated in genes related to fatty acid transport in HD mice (Fig. 5D and supplemental Fig. S2A). Furthermore, heatmap analysis of lipid metabolism-related genes showed that the expression of lipogenic genes was markedly enhanced in the HD mice, whereas Genes for lipid oxidation were markedly enhanced in HN mice (Fig. 5D and supplemental Fig. S2A). Thus, high-fat DRF mice may lead to hepatic lipid hyperplasia by promoting lipogenesis, while high-fat NRF mice reduce hepatic lipid deposition by promoting fat oxidation. The gene expression of lipid oxidation was enhanced in normal chow DRF mice compared with normal chow ad libitum fed mice, suggesting that the liver metabolizes more lipids through lipid oxidation.

To investigate the mechanism of hepatic lipid accumulation in high-fat DRF mice, we performed KEGG analysis on co-altered DEGs. Through KEGG analysis, we found that MAFLD-related pathways were enriched in both NCD and HFD backgrounds (Fig. 5E, F). We focused on the changes in *Cd36, Srebp*, and AMPK in the MAFLD-related pathway (supplemental Fig. S2B). Compared with HA mice, the expression of Cd36 and Nuclear *Srebp2* (N-SREBP2) in the MAFLD-related pathway was significantly increased in HD mice. In addition, Western blotting (WB) analysis showed that the expression of MAFLD signaling molecules *Cd36* and N-SREBP2 in HD mice was increased, and AMPK phosphorylation was decreased (Fig. 5H and supplemental Fig. S2D). From the above results, we found that DRF inhibits phosphorylation of AMPK, but some studies have shown that starvation signaling can activate AMPK phosphorylation (40, 41), which may explain the lipid balance in the liver of normal chow DRF mice (Fig. 5G and supplemental Fig. S2C). In high-fat DRF mice, *Cd36* expression increased dramatically, and phosphorylation of AMPK was significantly inhibited. The balance of lipid synthesis and oxidation was disrupted, resulting in lipid deposition in the liver of mice.

### Cd36 liver-specific knockout alleviates lean MAFLD in high-fat DRF mice

**Fig. 6:**
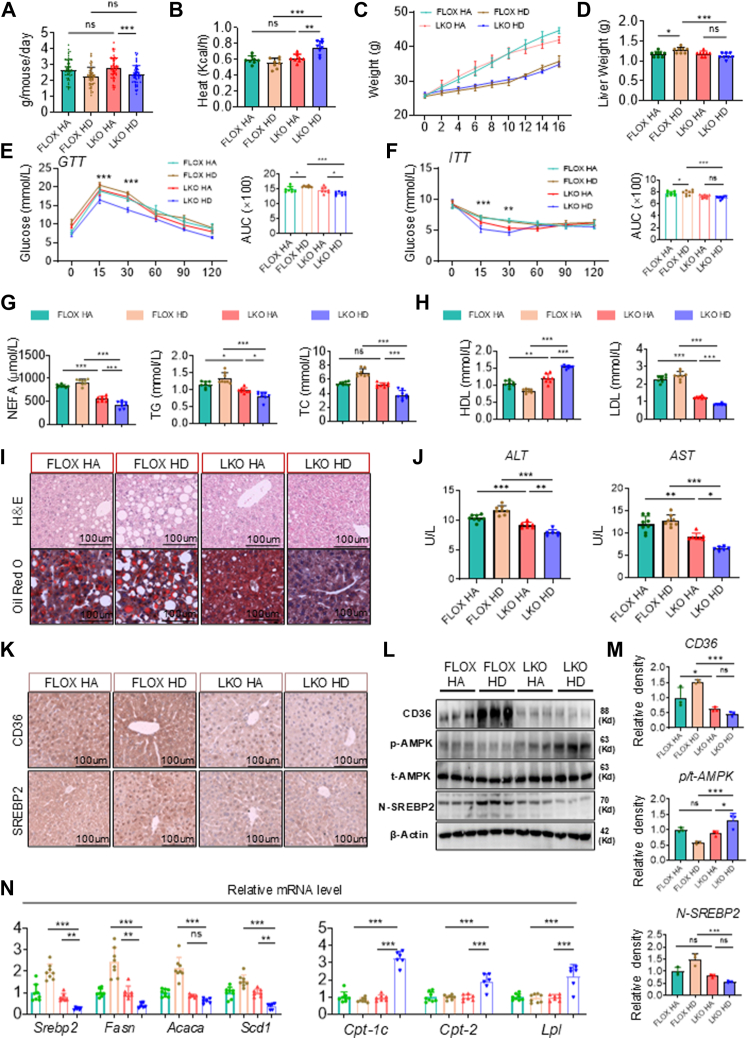
A–C: Daily intake, heat production per hour, and body weight of CD36flox/flox (FLOX) and CD36-LKO (LKO) mice in HFD background (n = 8). D: Liver weight of mice. E, F: GTTs and ITTs in FLOX and LKO mice. Quantification of the AUC was shown on the *right* (n = 8). G, H: Serum levels of TG, NEFA, TC, HDL, and LDL in FLOX and LKO mice (n = 8). I: Representative pictures of HE-staining and ORO-staining in mice liver sections were shown. J: the serum levels of AST and ALT in the mice (n = 6–8). K: Immunohistochemistry staining of CD36 and SREBP2 in the livers of FLOX and LKO mice. Scale bars = 100 μm. L: The protein levels of CD36, p-AMPK, t-AMPK, and N-SREBP2 in livers from the FLOX and LKO mice. M: Relative densities of CD36, AMPK, and N-SREBP2 proteins in Figure 6L. N: Relative mRNA levels of lipid synthesis (Srebp2, Fasn, Acaca, Scd1) and lipid oxidation (Cpt1, Cpt2, Lpl) in mice liver (n = 6–8). Statistical data were assessed using 1-way ANOVA with Tukey's multiple comparisons test. Data are presented as mean ± SEM. ∗*P* < 0.05; ∗∗*P* < 0.01; ∗∗∗*P* < 0.001.

**Figure S3:**
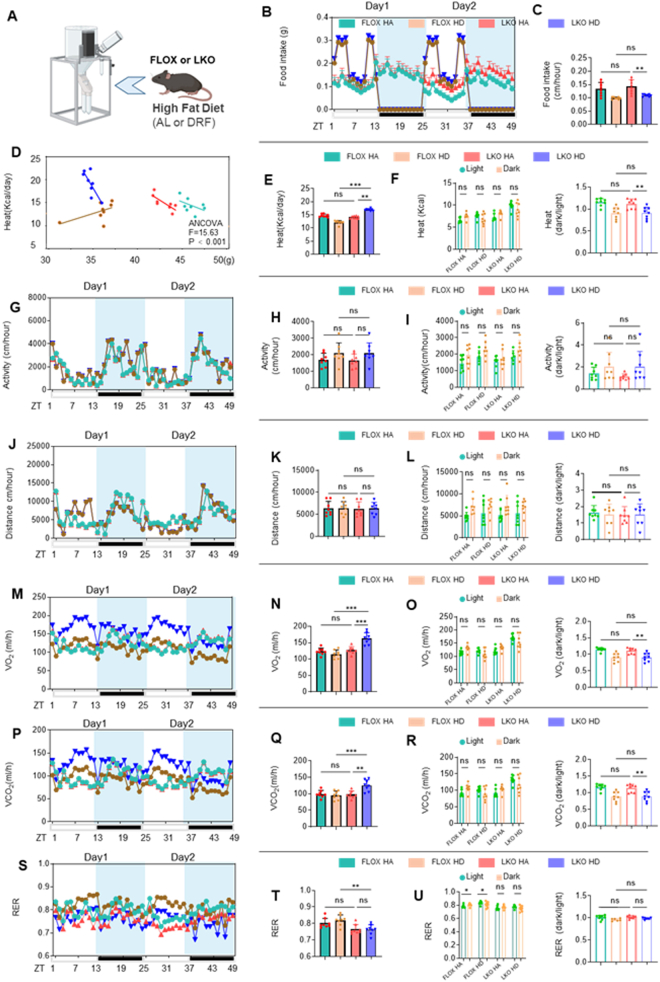
(A) Schematic of a mouse in a metabolic cage. (B-C) Food consumption per hour across light/dark phases and mean hourly activity in ad libitum-fed or DRF FLOX [or LKO] mice on HFD. (D-F) Heat production per hour in the light/dark cycle, average heat production per hour, and dark/light heat production ratio in ad libitum-fed or DRF FLOX [or LKO] mice on HFD (n=6-8/group, recorded for 2 days). (G-I) Locomotor. (J-L) Movement distance. (M-O) O2 consumption. (P-R) CO2 consumption. (S-U) RER statistical data（(n=6-8/group, recorded for 2 days). Statistical data were assessed using 1-way ANOVA with Tukey's multiple comparisons test or ANCOVA with body weight as the covariate when appropriate. Data are presented as mean ± SEM. ∗P < 0.05; ∗∗P < 0.01; ∗∗∗P < 0.001.

To evaluate the role of *Cd36* in the pathogenesis of lean MAFLD induced by high-fat dietary restriction-feeding (DRF), we generated liver-specific *Cd36* knockout (LKO) mice by crossing CD36 fl/fl (FLOX) mice with Albumin-cre mice. Both FLOX and LKO mice were subjected to either ad libitum feeding or HFD DRF for 16 weeks. Metabolic cage analysis (supplemental Fig. S3A) revealed that while LKO mice maintained similar daily food intake compared to control FLOX mice (Fig. 6A and supplemental Fig. S3B, C), they exhibited increased heat production (Fig. 6B and supplemental Fig. S3D–F). Notably, despite comparable locomotor activity between groups (supplemental Fig. S3G–L), LKO HD mice demonstrated elevated VO_2_ (supplemental Fig. S3M–O) and VCO_2_ (supplemental Fig. S3P–R), with a more pronounced increase in VO_2_. This led to a disrupted RER (VCO_2_/VO_2_) rhythm (supplemental Fig. S3S–U), with no significant difference in the diurnal RER variation between LKO HA and LKO HD mice (supplemental Fig. S3U). Following hepatic *Cd36* deletion, HD mice showed no significant change in body weight but displayed a marked reduction in liver weight (Fig. 6C, D). Compared to FLOX HD mice, LKO HD mice exhibited improved glucose tolerance and enhanced insulin sensitivity (Fig. 6E, F). Serum analysis further revealed that hepatic *Cd36* knockout significantly reduced circulating levels of TG, TC, NEFA, and LDL while increasing HDL in HD mice (Fig. 6G, H), indicating amelioration of DRF-induced dyslipidemia and insulin resistance. Histological assessment demonstrated decreased lipid accumulation in LKO HD mice, as evidenced by Oil Red O staining (Fig. 6I). And serum ALT and AST levels were significantly lower in LKO HD mice (Fig. 6J). These findings collectively suggest that liver-specific *Cd36* ablation mitigates metabolic dysregulation and hepatic steatosis in high-fat DRF-fed mice.

Based on transcriptomic data, we hypothesized that the increased *Cd36* in the liver of high-fat DRF mice was responsible for the inhibition of AMPK phosphorylation and increased expression of *N-Srebp2*. To verify this conjecture, we first performed immunohistochemical tests on the livers of mice in each group and found that the expression of *Cd36* and *N-Srebp2* in LKO HD mice was reduced (Fig. 6K). Second, WB results revealed enhanced AMPK phosphorylation and decreased N-SREBP2 expression in LKO HD mice (Fig. 6L, M). The Q-PCR results showed that LKO HD mice had upregulated lipid oxidation genes and downregulated lipid synthesis genes compared to FLOX HD mice (Fig. 6N). Therefore, these data indicated that *Cd36* plays an important role in the lean MAFLD induced by the high-fat DRF, and that the liver knockout of *Cd36* can enhance the phosphorylation of AMPK and decrease *N-Srebp2* expression to alleviate lean MAFLD in mice.

### SiCd36 mRNA liposomal nanoparticles alleviate lean MAFLD in high-fat DRF mice

**Fig. 7:**
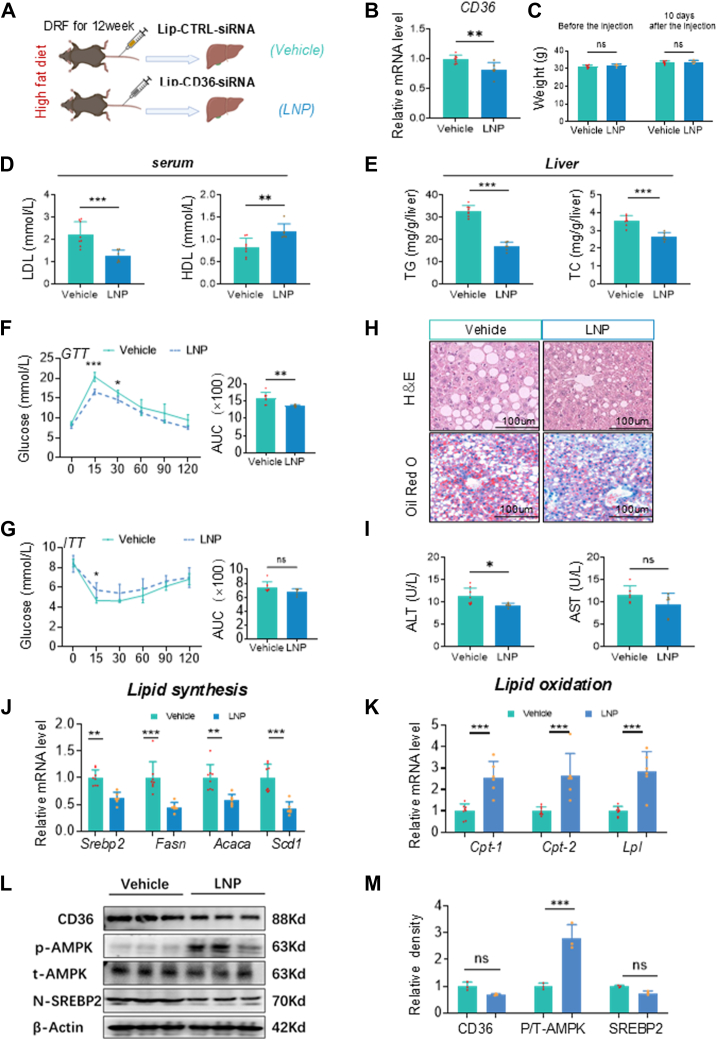
A: Schematic outline of mice HD for 12 weeks and injected with liposomal nanoparticles (LNP) via tail vein. B: Relative mRNA levels of CD36 (n = 6–8). C: Body weight before injection and 10 days after injection (n = 6–8). D: Serum levels of LDL and HDL (n = 6–8). E: Hepatic TG and TC content of livers (n = 6–8). F, G: GTTs and ITTs in vehicle and LNP mice (n = 6–8). H: Representative pictures of HE-staining and ORO-staining in mice liver sections were shown. I: Serum levels of ALT and AST (n = 6–8). J, K: Relative mRNA levels of lipid synthesis (Srebp2, Fasn, Acaca, Scd1) and lipid oxidation (Cpt1, Cpt2, Lpl) in mice liver (n = 6–8). L, M: The protein levels of CD36, p-AMPK, t-AMPK, and N-SREBP2 in livers and relative densities of CD36, AMPK, and N-SREBP2 proteins. Statistical data were assessed using 1-way ANOVA with Tukey's multiple comparisons test. Data are presented as mean ± SEM. ∗*P* < 0.05; ∗∗*P* < 0.01; ∗∗∗*P* < 0.001.

**Figure S4:**
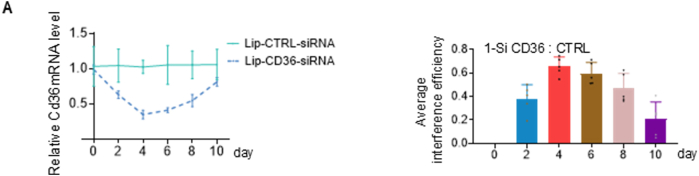
(A) Time-course analysis of CD36 mRNA expression following transfection with SiCD36-loaded Lipo3000 (0-10 days). Silencing efficiency was determined by qRT-PCR and normalized to day 0 control (n=5 independent experiments).

In recent years, lipid nanoparticles have been developed to facilitate both passive and active drug targeting of the liver, establishing them as a relatively mature targeted delivery system (42, 43). Due to the absence of a specific inhibitor for *Cd36*, we aimed to validate the potential of *Cd36* intervention for targeted therapy in lean MAFLD and to assess the effects of *Cd36* intervention during the progression of lean MAFLD. In vitro siRNA-mediated knockdown experiments in HepG2 cells confirmed optimal silencing efficiency between days 2–6, with gradually diminishing effects thereafter (supplemental Fig. S4A). We synthesized SiCd36 mRNA liposomal nanoparticles (LNP) and injected it into mice after 12 weeks of high-fat DRF (44) (Fig. 7A). Liver tissues were collected 10 days post-injection. There was no significant change in body weight after injection compared to the control group (vehicle) (Fig. 7C), and through the Q-PCR results, we found that *Cd36* mRNA expression was reduced (Fig. 7B). This proves that the injections are effective. LDL decreased and HDL increased in the serum of LNP mice (Fig. 7D). Glucose intolerance and decreased insulin sensitivity are relieved (Fig. 7F, G), indicating that dyslipidemia and glucose metabolism disorders were improved in LNP mice. In addition, TG and TC in the liver of mice are reduced (Fig. 7E). Oil red O staining revealed less lipid accumulation in the livers of LNP mice than those of Vehicle mice (Fig. 7H). It indicated that hepatic lipid accumulation caused by high-fat DRF was alleviated. At the same time, ALT and AST in the serum are reduced (Fig. 7I). This demonstrates that hepatic lipid accumulation in high-fat DRF mice was alleviated after injection. The results of QPCR showed that the expression of lipid synthesis genes was downregulated, and the expression of lipid oxidation genes was up-regulated in LNP mice (Fig. 7J, K). We further confirmed these results in the livers by immunoblot analysis. WB showed decreased expression of CD36 and N-SREBP2 protein and increased phosphorylation of AMPK in LNP mice (Fig. 7L, M). Therefore, these results demonstrated the role of targeted intervention *Cd36* in the course of lean MAFLD.

## Discussion

In this study, we investigated the relationship between dietary patterns and liver health. Mouse model effectively recapitulates key features of human metabolic disorders while validating the therapeutic benefits of TRF. At the molecular level, HFD-NRF mice exhibited significant upregulation of hepatic fatty acid oxidation genes (particularly *CPT1A*), reflecting the characteristic gene expression patterns observed in clinical studies of TRF-induced improvement in hepatic steatosis (45, 46). Phenotypically, these mice showed substantial improvements in glucose tolerance and insulin sensitivity, consistent with multiple clinical reports documenting TRF's metabolic advantages (47, 48). Notably, we observed significant amelioration of the lipid profile in HFD-NRF mice, including reduced triglycerides and LDL-cholesterol levels coupled with elevated HDL-cholesterol. These findings further corroborate the beneficial effects of TRF on lipid metabolism, as evidenced by human clinical studies (49, 50).

Daytime-restricted feeding in nocturnal mice (where food is available only during their inactive phase) corresponds to nighttime-restricted feeding in diurnal humans (where food is consumed during their inactive phase) Contrary to previous findings that suggest time-restricted feeding ameliorates metabolic diseases, we discovered that reversing dietary timing can introduce additional metabolic risks to the body, increasing susceptibility to lipid metabolic diseases. Notably, high-fat DRF led to the development of lean MAFLD in mice, a phenomenon that has not been previously reported. Contrary to previous reports suggesting that CD36 deficiency might have certain adverse effects, our findings demonstrate that CD36 deficiency can confer resistance to the metabolic risks induced by high-fat DRF. This suggests that CD36-deficient individuals may possess enhanced resilience against metabolism-related disorders caused by altered feeding schedules. Furthermore, we elucidated the relationship between DRF and lean MAFLD, identifying that the primary mechanism underlying DRF-induced lean MAFLD is the upregulation of CD36 expression in the liver. CD36 is a multifunctional protein involved in the regulation of lipid metabolism and had not previously been associated with lean MAFLD. By targeting interventions in liver CD36 expression with CD36 liver-specific knockout and liposomal nanoparticles, we established CD36 as a potential therapeutic target for interventions targeting lean MAFLD.

In recent years, dietary interventions, such as time-restricted feeding, intermittent fasting, and fasting mimicking diets, have gained popularity as potential strategies against metabolic diseases (51, 52). However, certain populations, such as night workers (17) and fasting Muslims (18), may not always align their eating patterns with the body’s circadian rhythm. This misalignment has been associated with an increased risk of metabolic disorders; The underlying mechanisms remain unclear and may involve disruptions in multiple metabolic pathways (53). Notably, existing studies have primarily concentrated on the influence of the biological clock on metabolic processes, often neglecting the potential impact of dietary factors. Our study on mice revealed that feeding at inappropriate times resulted in elevated blood lipids, indicating an increased metabolic risk. In the context of NCD, DRF caused a slight increase in CD36 expression, which was counteracted by the effects of DRF, leaving the mice in a state of starvation. However, when combined with HFD, the significant elevation of CD36 expression in mice resulted in the accumulation of lipids in the liver, despite the overall low energy intake of the body, preventing obesity.

The RER rhythm reflects the pattern of whole-body fuel utilization, which is an overall product of a glucose utilization rhythm (peaking during the fed state) and a fat utilization rhythm (peaking during the fasted state) (16, 27). Ad libitum and NRF mice typically exhibit parallel rhythms of RER, while DRF mice exhibit strong RER rhythms. This illustrates the alteration of the circadian rhythm of glucose and lipid metabolism in DRF mice. In addition, DRF mice consistently showed a steeper decline in RER during fasting. This not only reflects the increased utilization of lipids as an energy source (16, 27) but also shows that more lipids are being transported to the liver.

By analyzing the RNA sequences, it was observed that DRF led to a significant increase in lipid synthesis genes in mice, particularly in the presence of an HFD. Lipid oxidation genes were inhibited in high-fat DRF mice. However, in the context of an NCD, lipid oxidation genes were enhanced, indicating that mice metabolize synthetic lipids through oxidation. This is consistent with the absence of clinical symptoms in early lean MAFLD patients (54, 55, 56), suggesting metabolic disturbances in the liver. The dysregulation of lipid metabolism induced by DRF may be attributed to various factors, with a focus on lipid transport, synthesis, and oxidation. Bioinformatics analysis highlighted CD36, SREBP, and AMPK as potential targets. The significance of CD36 in the pathogenesis of MAFLD has been indicated. Studies have shown that CD36 expression is upregulated in the liver with steatosis compared to healthy livers (57). In our study, we observed elevated CD36 expression in DRF mice, particularly in the presence of a high-fat diet. In this study, we observed a decrease in AMPK phosphorylation and an increase in N-SREBP2 expression in high-fat DRF mice compared to control high-fat ad libitum feeding mice. This indicates that, despite the enhanced transport of lipids to the liver, lipid oxidation was inhibited, leading to an untimely utilization of these lipids. Consequently, this results in the accumulation of lipids within the liver.

In light of these, we guessed that CD36 was required to cause high-fat DRF-induced lean MAFLD in mice. Therefore, we used CD36 liver-specific knockout mice to explore the role of CD36 in lean MAFLD. Consistent with previous reports, the expression of N-SREBP2 was significantly decreased in high-fat DRF mice after CD36 liver-specific knockout, while AMPK phosphorylation in liver mitochondria was markedly increased. In addition, we also found that the disturbance of glucose and lipid metabolism induced by high-fat DRF disappeared in mice after liver-specific knockout of CD36. These results demonstrated that CD36 is a key factor in high-fat DRF-induced lean MAFLD. The absence of an active pocket in the CD36 structure has posed a challenge in identifying specific inhibitors for this protein (58). To address this issue and inhibit CD36 expression in mouse disease models, we developed SiCD36 mRNA liposomal nanoparticles. Administration of these nanoparticles via tail vein injection in mice at the 12th week of high-fat DRF-induced lean MAFLD resulted in improvements in liver lipid accumulation. Given these findings, individuals with CD36 deficiency may exhibit greater adaptability to inverted dietary patterns and night shift work. However, the similarities between different species warrant further investigation.

Although this study demonstrates that CD36 knockout significantly inhibited high-fat DRF-induced lean MAFLD, it has certain limitations. Firstly, while we observed a significant impact of a 12-h DRF on hepatic metabolism, we did not investigate the effects of other feeding windows, such as 4-h DRF or 8-h DRF, on liver metabolism (35). Secondly, CD36 exhibits rhythmic expression in the liver (59). We reduced CD36 expression through liposomal nanoparticles intervention, but we did not employ temporal therapy to modulate CD36 (60, 61, 62). We primarily elucidated its function as a fatty acid translocase, and future studies will continue to explore the implications of its rhythmic function. Nevertheless, our current findings strongly support the observation that metabolic disorders in mice are significantly alleviated following liposome injection. Thirdly, this study exclusively used male mice, which overlooks potential sexual dimorphism in metabolic responses. Prior research indicates that young female mice may exhibit distinct metabolic adaptations to high-fat diets due to estrogen-mediated protection, whereas aging females show increased susceptibility to metabolic dysfunction (63, 64). Future studies should include both sexes to better evaluate sex- and age-dependent effects. Nevertheless, our current findings strongly support the observation that metabolic disorders in mice are significantly alleviated following liposome injection. Lastly, this study did not include safety and targeting experiments for SiCd36 mRNA Liposomal nanoparticles, which would strengthen our findings. Although research in this area has been addressed in other publications (65, 66).

In conclusion, DRF increases the susceptibility to lipid metabolism disorders, and high-fat DRF induces lean MAFLD. CD36 has been identified as a critical mediator in the pathogenesis of lean MAFLD induced by dietary factors, primarily through its role in inhibiting phosphorylation of AMPK, thereby disrupting the equilibrium between lipid synthesis and oxidation. This study underscores the potential of CD36 as a therapeutic target for addressing lean MAFLD associated with high-fat DRF. Furthermore, it offers valuable insights into interventions and treatments for metabolic disorders arising from dietary influences, particularly in populations subjected to night shifts or fasting conditions.

## Data availability

Data are available on reasonable request to the corresponding author. The raw RNA-seq data have been deposited in the NCBI SRA under accession number PRJNA1234793.

## Conflict of interest

The authors declare that they have no conflicts of interest with the contents of this article.

## Figures and Tables

**Figure S5:**
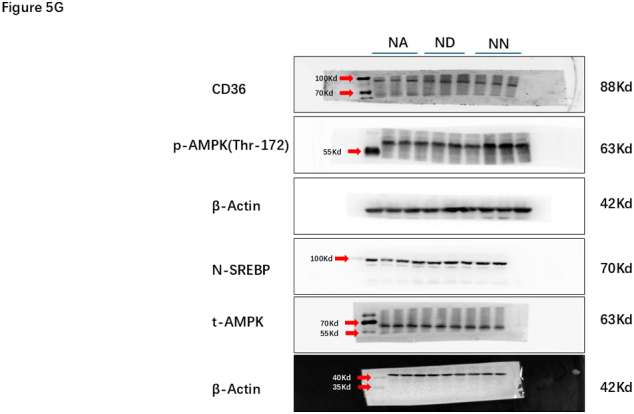
Original WB blots of Figure 5G

**Figure S6:**
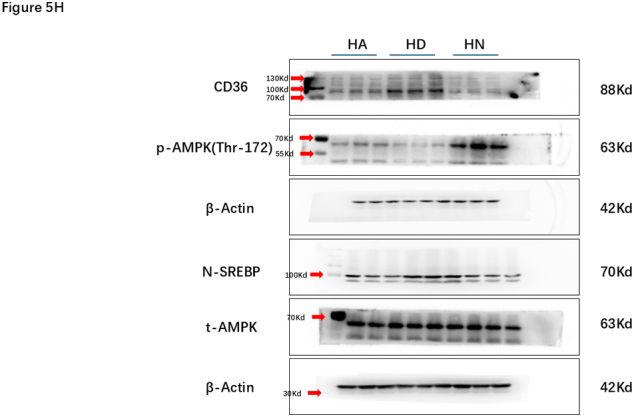
Original WB blots of Figure 5H

**Figure S7:**
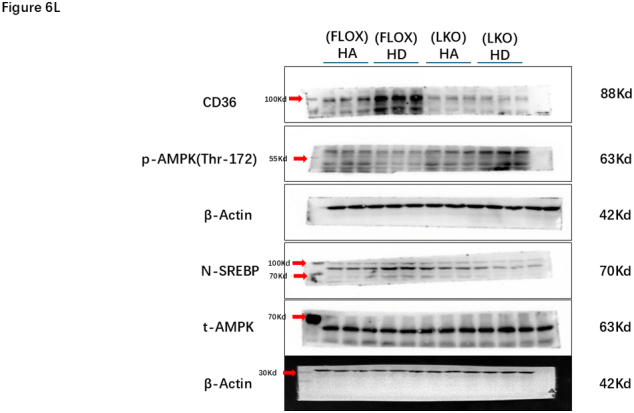
Original WB blots of Figure 6L

**Figure S8:**
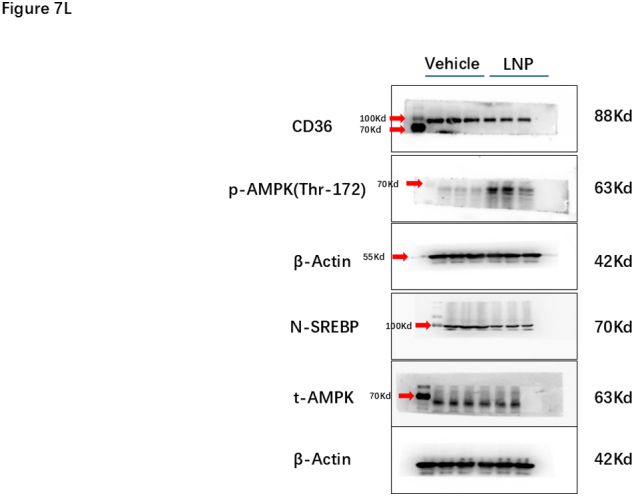
Original WB blots of Figure 7L

## Supplemental Data

Information of primary antibodies

Primers for quantitative real-time PCR
