# Supplementary material for: Daytime-restricted feeding induces lean MAFLD in high-fat diet-fed mice by upregulating CD36-mediated lipid accumulation
Source: J Lipid Res. 2025 Jun 23;66(8):100853. doi: 10.1016/j.jlr.2025.100853 (PMC12302294; doi:10.1016/j.jlr.2025.100853)
Supplement: Information of primary antibodies [file mmc1.docx]

**Supplemental table S1: Information of primary antibodies**

| Antibodies | Species | Dilution ratio | brand | Catalog number |
| --- | --- | --- | --- | --- |
| **CD36** | Rabbit | 1:2000 (WB)  1:250(IHC) | Novus | NB400-144 |
| **SREBP1** | Rabbit | 1:2000 (WB)  1:300(IHC) | Abcam | 28481 |
| **t-AMPK** | Rabbit | 1:2000 (WB) | Cell Signaling | 2532 |
| **P-AMPK(Thr172)** | Rabbit | 1:1000 (WB) | Cell Signaling | 2535 |
| **β-actin** | Rabbit | 1:10000 (WB) | Bioss | 0061R |
