# Supplementary material for: Daytime-restricted feeding induces lean MAFLD in high-fat diet-fed mice by upregulating CD36-mediated lipid accumulation
Source: J Lipid Res. 2025 Jun 23;66(8):100853. doi: 10.1016/j.jlr.2025.100853 (PMC12302294; doi:10.1016/j.jlr.2025.100853)
Supplement: Primers for quantitative real-time PCR [file mmc2.docx]

**Supplemental table S2: Primers for quantitative real-time PCR**

| Genes | Forward primer | Reverse primer |
| --- | --- | --- |
| CD36 | ATGGGCTGTGATCGGAACTG | GTCTTCCCAATAAGCATGTCTCC |
| FATP4 | CATCTACACATCGGGCACC | GTCATGCCGTGGAGTAAGCA |
| FATP5 | CAGAGGGCAATGTGGGCTTAAT | TCTGCTGTCTCTATGTCGAACTG |
| SREBP1 | ATGCGGCTGTTGTCTACCATAAG | CCACATAGATCTCTGCCAGTGTT |
| FASN | CCTTCCGTCACTTCCAGTTAGAG | AAGTTCAGTGAGGCGTAGTAGAC |
| SCD1 | CATCGCCTGCTCTACCCTTTAAA | GTGGTCGTGTAAGAACTGGAGAT |
| ACACA | GCTGGGACTGTGGAATACTTGTA | GGCAAGGAACATGAGCAGAATTT |
| Il-6 | GCTACCAAACTGGATATAATCAGGAAA | CTTGTTATCTTTTAAGTTGTTCTTCATGTACTC |
| Il-1β | GCAACTGTTCCTGAACTCAACT | ATCTTTTGGGGTCCGTCAACT |
| Tnf-α | CCCTCACACTCAGATCATCTTCT | GCTACGACGTGGGCTACAG |
| CPT1 | GCAAATGATGTGGACCTGCATTC | AGAACTTGCCCATGTCCTTGTAA |
| CPT2 | TATTCGCCCAGCTTCCATCTTTA | GCCAGATACCGTAGAGCAAACAA |
| LPL | TCTGTGTCTAACTGCCACTTCAA | GGCCCGATACAACCAGTCTACTA |
